# How to Arrange Follow-Up Time-Intervals for Longitudinal Brain MRI Studies in Neurodegenerative Diseases

**DOI:** 10.3389/fnins.2021.682812

**Published:** 2021-07-15

**Authors:** Hans-Peter Müller, Anna Behler, G. Bernhard Landwehrmeyer, Hans-Jürgen Huppertz, Jan Kassubek

**Affiliations:** ^1^Department of Neurology, University of Ulm, Ulm, Germany; ^2^Swiss Epilepsy Clinic, Klinik Lengg, Zurich, Switzerland

**Keywords:** magnetic resonance imaging, longitudinal study, time-interval, linear fit, regression analysis

## Abstract

**Background:**

Longitudinal brain MRI monitoring in neurodegeneration potentially provides substantial insights into the temporal dynamics of the underlying biological process, but is time- and cost-intensive and may be a burden to patients with disabling neurological diseases. Thus, the conceptualization of follow-up time-intervals in longitudinal MRI studies is an essential challenge and substantial for the results. The objective of this work is to discuss the association of time-intervals and the results of longitudinal trends in the frequently used design of one baseline and two follow-up scans.

**Methods:**

Different analytical approaches for calculating the linear trend of longitudinal parameters were studied in simulations including their performance of dealing with outliers; these simulations were based on the longitudinal striatum atrophy in MRI data of Huntington’s disease patients, detected by atlas-based volumetry (ABV).

**Results:**

For the design of one baseline and two follow-up visits, the simulations with outliers revealed optimum results for identical time-intervals between baseline and follow-up scans. However, identical time-intervals between the three acquisitions lead to the paradox that, depending on the fit method, the first follow-up scan results do not influence the final results of a linear trend analysis.

**Conclusions:**

This theoretical study analyses how the design of longitudinal imaging studies with one baseline and two follow-up visits influences the results. Suggestions for the analysis of longitudinal trends are provided.

## Introduction

There is a constantly growing number of longitudinal neuroimaging studies in neurological and particularly neurodegenerative diseases over the last 15 years. Longitudinal brain MRI studies in neurology are time- and cost-intensive and may be a burden to patients with disabling diseases. Thus, the schedule of longitudinal time-intervals is an essential aspect in the conceptualization of longitudinal MRI studies and may be substantial for the validity of the results. Compared to cross-sectional data, the longitudinal design can provide increased statistical power by reducing the confounding effect of between-subject variability (Thompson et al., 2011). Furthermore, longitudinal studies provide unique insights into the temporal dynamics of the underlying biological process (Sabuncu et al., 2011; Jack et al., 2012); a serial assessment can be the only way to unambiguously characterize the effect of interest in a randomized experiment such as a pharmacological clinical trial (Davis et al., 2005; Dickerson and Sperling, 2005).

In the domain of clinical neuroimaging, the design of longitudinal studies with respect to the schedule of the follow-ups is not widely discussed and in practice is subject to the limitations imposed by participants as well as financial considerations of the study. However, there are approaches to longitudinal study design from study targets such as aging processes (Newman, 2010). As the strategic planning of a longitudinal study includes dimensions such as the hypothesized true variance in change, indicator reliability, the number and spacing of measurement occasions, total study time, and sample size, the main search goal is to select a research design that best addresses the guiding questions and hypotheses of the planned study while heeding applicable external conditions and constraints, including time, money, feasibility, and ethical considerations. Brandmaier et al. (2015) proposed the Longitudinal Interactive Front End Study Planner (LIFESPAN) framework to generate a set of alternative models with equal statistical power to detect hypothesized effects and delineates trade-off relations among relevant parameters.

In longitudinal MRI studies of neurodegenerative diseases, due to a balance between costs and timeline and the validity of the results, a common concept is to plan with one baseline and two follow-up studies with (almost) identical time intervals, i.e., bisectioning the observation time period. Examples for such a study design can be found in amyotrophic lateral sclerosis (ALS) (Cardenas-Blanco et al., 2016; Kassubek et al., 2018; Kalra et al., 2020; Steinbach et al., 2021; Tahedl et al., 2021), in Huntington’s disease (Tabrizi et al., 2009; Hobbs et al., 2015; Müller et al., 2019). This study design usually allows for a linear fit to obtain longitudinal trends of resulting parameters in the respective imaging domain. Here, we theoretically investigated how the fit methods of linear regression analysis and the choice of time intervals influence the final results for longitudinal trends of a given (arbitrary) parameter of interest. We expected the results of this investigation to facilitate the scheduling of acquisition time points for upcoming studies and to guide in calculating linear trends for already ongoing studies with three measurement time points and identical intervals.

## Methods

Several fit methods for calculating the linear trend of longitudinal parameters were tested analytically and in simulations, with special respect on their performance to deal with the effect of outliers.

A linear trend analysis of *N* data points (*t_i_*; y_*i*_) follows:

(1) y=I⁢t+b

with inclination *I* and intercept *b*.

We specifically investigated the frequently used design of three acquisition time points (see e.g., Cardenas-Blanco et al., 2016; Tahedl et al., 2021), i.e., time point 1 at baseline and time points 2 and 3 at follow-up 1 and 2, respectively.

### Models for Trend Analysis

**FIGURE 1:**
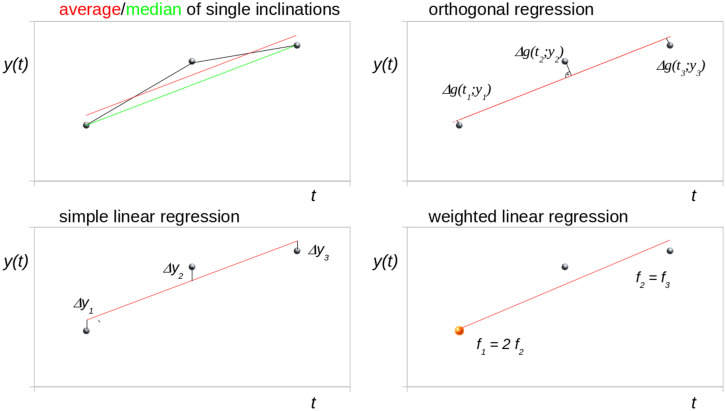
Schematic illustration of different trend analysis approaches for the fit of three data points for identical time intervals (bisectioning the observation time). The central data point was shifted to simulate a disturbance. *Δg* denotes the orthogonal distance to the regression line.

Figure 1 schematically illustrates different trend analysis approaches for the fit of three data points for identical time intervals, i.e., bisectioning the observation time period. The data point at *t*_2_ was offset by an arbitrary amount from the modeled linear trend to simulate a disturbance (e.g., a measurement affected by motion artifacts). Possible trend analysis approaches are listed below, following (Rencher and Schaalje, 2007):

#### Average of Single Inclinations

The linear longitudinal trend of an arbitrary parameter can be calculated from the arithmetic average of the single inclinations between two time points (*I_12_, I_23_, I_13_*). For three time points (*t_1_, t_2_, t_3_*), the mean inclination *T*_*avg*_ is calculated as

$$T_{a\,v\,g}=\frac{1}{3}\,\left(I_{12}+I_{23}+I_{13}\right)$$

(2) $$=\frac{1}{3}\,\left[\left(\frac{y_{1}-y_{2}}{t_{1}-t_{2}}\right)+\left(\frac{y_{2}-y_{3}}{t_{2}-t_{3}}\right)+\left(\frac{y_{1}-y_{3}}{t_{1}-t_{3}}\right)\right]$$

As for any arithmetic average, the values to be averaged should be normally distributed.

#### Median of Single Inclinations

In the case of non-normal distribution, however, the median value can be used instead of the arithmetic average to estimate the overall linear trend:

$$T_{m\,e\,d\,i\,a\,n}=M\,E\,D\,I\,A\,N\,\left[I_{1};I_{2};I_{3}\right]$$

(3) $$=M\,E\,D\,I\,A\,N\,\left[\left(\frac{y_{1}-y_{2}}{t_{1}-t_{2}}\right);\left(\frac{y_{2}-y_{3}}{t_{2}-t_{3}}\right);\left(\frac{y_{1}-y_{3}}{t_{1}-t_{3}}\right)\right]$$

#### Simple Linear Regression

An advanced approach to estimate the overall trend by fitting a straight line using a least-squares approach is presented in Press et al. (1992a). In this approach, the sum of squared differences *d_*i*_^2^* of *y*-values to the regression line is minimized:

(4) $$m\,i\,n\,\sum_{i=1}^{N}d_{i}^{2}=m\,i\,n\,\sum_{i=1}^{N}{\left(y_{i}-\left(I\,t_{i}+b\right)\right)}^{2}$$

The general formula for calculating the overall trend then reads as follows:

(5) $$T_{l\,i\,n\,R\,e\,g}=\frac{\sum_{i=1}^{N}u_{i}\,v_{i}}{\sum_{i=1}^{N}u_{i}^{2}}=\frac{W}{U}$$

where *u*_*i*_ and *v*_*i*_ denote the differences to the mean in time and the mean in *y*-values, respectively:

$$u_{i}=t_{i}-\bar{t};\bar{t}=\frac{1}{N}\,\sum_{i=1}^{N}t_{i}$$

$$v_{i}=y_{i}-\bar{y};\bar{y}=\frac{1}{N}\,\sum_{i=1}^{N}y_{i}$$

The terms composed of *u*_*i*_ and *v*_*i*_ can be abbreviated to:

(6) $$U=\sum_{i=1}^{N}u_{i}^{2}\;V=\sum_{i=1}^{N}v_{i}^{2}\;W=\sum_{i=1}^{N}u_{i}\,v_{i}$$

This prepares subsequent Eq. 8 in the orthogonal regression approach.

For three time points, the trend *T*_*linReg*_ is calculated as:

(7) $$T_{l\,i\,n\,R\,e\,g}=\frac{\left(t_{1}-\bar{t}\right)\,\left(y_{1}-\bar{y}\right)+\left(t_{2}-\bar{t}\right)\,\left(y_{2}-\bar{y}\right)+\left(t_{3}-\bar{t}\right)\,\left(y_{3}-\bar{y}\right)}{{\left(t_{1}-\bar{t}\right)}^{2}+{\left(t_{2}-\bar{t}\right)}^{2}+{\left(t_{3}-\bar{t}\right)}^{2}}$$

If the measurement errors are normally distributed, then this fitting function will give maximum likelihood parameter estimations of the overall linear trend; if the errors are not normally distributed, then the estimations are not maximum likelihood, but may still be useful in a practical sense (Press et al., 1992a).

#### Orthogonal Regression

In the orthogonal regression, the squared *orthogonal* differences of data points to the fitted line are minimized; Eq. 4 changes to

(8) $$m\,i\,n\,\sum_{i=1}^{N}d_{i}^{2}=m\,i\,n\,\sum_{i=1}^{N}\frac{{\left(y_{i}-\left(I\,t_{i}+b\right)\right)}^{2}}{1+I^{2}}$$

and Eq. 5, thus, changes to

(9) $$T_{o\,r\,t\,h\,o\,R\,e\,g}=\frac{V-U+\sqrt{{\left(V-U\right)}^{2}+4\,W^{2}}}{2\,W}$$

The difference to *T*_*linReg*_ is that this approach minimizes not only the distances of *y*_*i*_ but also the distances of *t*_*i*_ to the regression line.

#### Weighted Linear Regression

If the variance and/or the quality of the measurements is not equal, this knowledge can be incorporated into the regression via least squares weighting. For each data point, the squared difference (Eq. 4) is multiplied by a weighting factor f_*i*_ prior to minimization:

(10) $$m\,i\,n\,\sum_{i=1}^{N}d_{i}^{2}=m\,i\,n\,\sum_{i=1}^{N}f_{i}\,{\left(y_{i}-\left(I\,t_{i}+b\right)\right)}^{2}$$

In general, the respective weights are the reciprocal variance of the measurements so that values with low variance are given higher weights and values with higher variance are given lower weights.

#### Fit Approach by Linear Mixed Effects Models

There are generally three potential sources of variability which influence the correlation structure in longitudinal data, i.e., (1) between-subject variation, (2) inherent within-subject biological change, and (3) measurement error (Fitzmaurice et al., 2011). Complex fit models for harmonizing longitudinal imaging data based on empirical Bayesian methods (combining batches – ComBat) (Beer et al., 2020), conditional growth models (Tahedl et al., 2021) or mixed effect models (Cardenas-Blanco et al., 2016) are used for analysis to take these influences into account. The linear mixed effects model for longitudinal data (Bernal-Rusiel et al., 2013) follows:

(11) $$y_{i}=x_{i}\,\beta +t_{i}\,u_{i}+e_{i}$$

where ***y****_*i*_* is the vector of longitudinal measurements for subject *i*, ***x****_*i*_* is the subject design matrix for the fixed effects (including clinical group, etc.), *β* is a vector of unknown fixed effects regression coefficients, ***t****_*i*_* is the design matrix for the random effects (e.g., scan time), ***u****_*i*_* is a vector of random effects, and ***e****_*i*_* is a vector of measurement errors, with constraints for the expectations

$$E\,\left(y_{i}\right)=x_{i}\,\beta$$

$$E\,\left(u_{i}\right)=0$$

(12) $$E\,\left(e_{i}\right)=0$$

The linear mixed effects model has a randomly varying intercept (*β_1_ + u_11_*) and slope (*β_2_ + u_21_*):

(13) $$y_{i\,j}=\left(\beta_{1}+u_{11}\right)+\left(\beta_{2}+u_{21}\right)\,t_{i\,j}+e_{i\,j}$$

where *y*_*ij*_ is the *j*-th measurement from subject *i*. The model of Eq. (13) allows each individual’s measurements to have a unique linear mean trend. A major advantage of this approach in detecting longitudinal group differences is that the subjects in the study are not required to have a common set of measurement time points (Bernal-Rusiel et al., 2013), i.e., time points could differ or even be missing. As a source of variation possibly influencing the longitudinal linear trend, between-subject variation does not play a role in the approach of the current study with three measurement time points from only one subject. Assuming that aberrations because of, for example, noisy measurements due to patient movements, have a much larger share in the change of the linear trend than inherent within-subject biological change, linear mixed effect models would not develop their full power in answering the question about the timing of only three measurements. In the simplified approach of the current study where only one data set (for one subject) with three timepoints was discussed, Eq. 13 reduces to

(14) $$y_{i}=\left(\beta_{2}+u_{1}\right)\,t_{i}+\left(\beta_{1}+u_{2}\right)$$

i.e., from three equations only *β_1_* and *β_2_* can be determined. Simplified linear mixed effects modeling was then realized by the application of a downhill simplex fitting routine (Press et al., 1992b).

(15) $$y_{i}=T_{f\,i\,t\,A\,p\,p\,r\,o\,a\,c\,h}\,t_{i}+b_{f\,i\,t\,A\,p\,p\,r\,o\,a\,c\,h}$$

### Simulations With Outliers and Time-Interval Dependency

For the concept of three measurement time points (one baseline and two follow-up scans) which is frequently used for longitudinal MRI studies in neurodegenerative diseases, a simulation with outliers/aberrations was performed, i.e., the effect of an artificial additive offset value at time points *t*_2_ and *t*_3_ was analyzed. The ensuing deviation of trend results compared to the original value of the overall trend (without any additive offset) was calculated.

In neurodegenerative diseases, at baseline (inclusion time) most participants are motivated and in a comparatively good physical condition. Thus, the time interval to follow-up is usually planned depending on the clinical evaluation of the disease progression, the estimation of the trend of a given parameter under observation, and the corresponding power calculations for the study. The reason for a second follow-up is often the interest in long-term observations and/or the intention to stabilize the trend results by another measurement; the time limitation here is the total duration of the planned study. However, during the course of the study, often the physical condition and also the motivation of the patients are worsening, leading to potentially reduced quality of scans at second follow-up.

In order to use concrete realistic numeric values we performed the data simulation based on the longitudinal striatum atrophy in MRI data of patients with Huntington’s disease; the underlying volumetric results were determined by atlas-based volumetry (ABV) of structural MRI data (Müller et al., 2019). ABV is an objective, investigator- independent technique with low intra-scanner variability to determine the volumes of intracranial compartments and cerebral substructures from the MRI data of individual subjects (Huppertz et al., 2010). Nevertheless, the results concerning time-interval planning and fit approach can be directly transferred to any MRI-based parameter in neurodegenerative diseases.

According to the results of the above-mentioned study in HD patients (Müller et al., 2019), a study period of 15 months and a monthly volume decrease of the striatum of 0.2% (in relation to its size at baseline) were assumed for our simulation. Numerical aberrations at time points *t*_2_ and *t*_3_ were simulated by a constant offset value of +0.3% (in relation to the striatum size at baseline, i.e., 8.3 cm^3^). The total study time (baseline to follow-up 2) was kept constant at 15 months in our simulation, but the follow-up 1 time was varied from 0 to 15 months and the aforementioned offset was applied.

(i) at *t*_2_ (follow-up 1) – simulating the situation of a high quality measurement at *t*_3_ and a measurement at *t*_2_ of limited quality.
(ii) at *t*_3_ (follow-up 2) – simulating the situation of a high quality measurement at *t*_2_ and a measurement at *t*_3_ of limited quality (corresponding to less patient motivation or worse patient condition).

## Results

### Analytical Calculation for Identical Time Intervals

For identical time intervals, i.e., bisectioning the observation time period yields

(16) $$t_{1}-t_{2}=t_{2}-t_{3}=\Delta \,t=\frac{1}{2}\,\left(t_{1}-t_{3}\right)$$

### Average of Single Inclinations

For identical time intervals, Eq. 2 simplifies to

$$T_{a\,v\,g}\,\left(\Delta \,t\right)=\frac{1}{3}\,\left[\frac{y_{1}-y_{2}}{\Delta \,t}+\frac{y_{2}-y_{3}}{\Delta \,t}+\frac{y_{1}-y_{3}}{2\,\Delta \,t}\right]$$

(17) $$=\frac{y_{1}-y_{3}}{2\,\Delta \,t}=\frac{y_{1}-y_{3}}{t_{1}-t_{3}}$$

That means that identical time-intervals have the effect that data points at *t*_2_ (follow-up 1) do not influence the overall trend.

#### Median of Single Inclinations

It is an intrinsic property for the median calculation Eq. 3 that the median is also independent of (*t_2_,y_2_*).

#### Simple Linear Regression

For identical time intervals $\left(\bar{t}=t_{2}\right)$, Eq. 7 simplifies to

(18) $$T_{l\,i\,n\,R\,e\,g}=\frac{\left(t_{1}-t_{2}\right)\,y_{1}+\left(t_{3}-t_{2}\right)\,y_{3}}{{\left(t_{1}-t_{2}\right)}^{2}+{\left(t_{3}-t_{2}\right)}^{2}}=\frac{\Delta \,t\,\left(y_{1}-y_{3}\right)}{2\,\Delta \,t^{2}}=\frac{y_{1}-y_{3}}{t_{1}-t_{3}}$$

Thus, in all three cases (average or median of inclinations, simple regression), identical time-intervals have the effect that data points at *t*_2_ (follow-up 1) do not influence the overall trend for either calculation method based on a linear model.

In general, the calculation of the slope for a linear regression simplifies for identical time intervals to:

(19) $$T=\frac{y_{1}-y_{3}}{t_{1}-t_{3}}$$

### Approaches Which Incorporate Timepoint 2 in the Trend Calculation

The other two approaches, i.e., orthogonal regression and weighted linear regression, theoretically take time point 2 (i.e., follow-up scan 1) into account when calculating the trend, but each have their own limitations.

#### Orthogonal Regression

For the case of three acquisitions with two identical time intervals, i.e., bisectioning the observation time period, the orthogonal regression theoretically incorporates the data point at *t*_2_ (follow-up scan 1). However, the variety between orthogonal regression and simple linear regression depends on the magnitude of the trend, i.e., the smaller the trend, the smaller is the variety between orthogonal regression and simple linear regression (both are equal for an inclination of 0). Given that the orthogonal regression is based on a minimization of *t*- and *y*-distances, however, a minimization of distances of *y*-values is usually preferred for a scenario of defined *t*- and measured *y*-values rather than a minimization of *t*-values (that way supporting the conditions of longitudinal MRI studies where the time points are exactly defined).

#### Weighted Linear Regression

The weighting of the different measurement points would, depending on the specific weighting factors, lead to consideration of the second time point, but would be a challenge, as the weighting has to be based on an independent parameter, e.g., a measurement quality control prior to the regression calculation. However, the weighted linear regression approach could be an option for specific conditions, e.g., when it is expected that the first measurement (when the patients are usually in a better condition compared to measurements at a later disease stage) will be of better quality than subsequent measurements. In this case, the weighting could be for example *f*_1_ = 1.5, and *f_2_ = f_3_* = 0.75 for the three visits, i.e., data points. However, the weighting is depending on the experimental design and needs a justification by the rater (based on data quality, patient condition, etc.). The justification of the weighting factors is a critical item. In order to use weighted linear regression, longitudinal studies could be planned in a way that the baseline results are stabilized by e.g., repeated scans at the baseline visit [in general, averaging within-session scans will improve between-session reliability (Lau and Goodyear, 2007)].

### Simulation of a Scenario of a Longitudinal Study in Neurodegenerative Diseases

**FIGURE 2:**
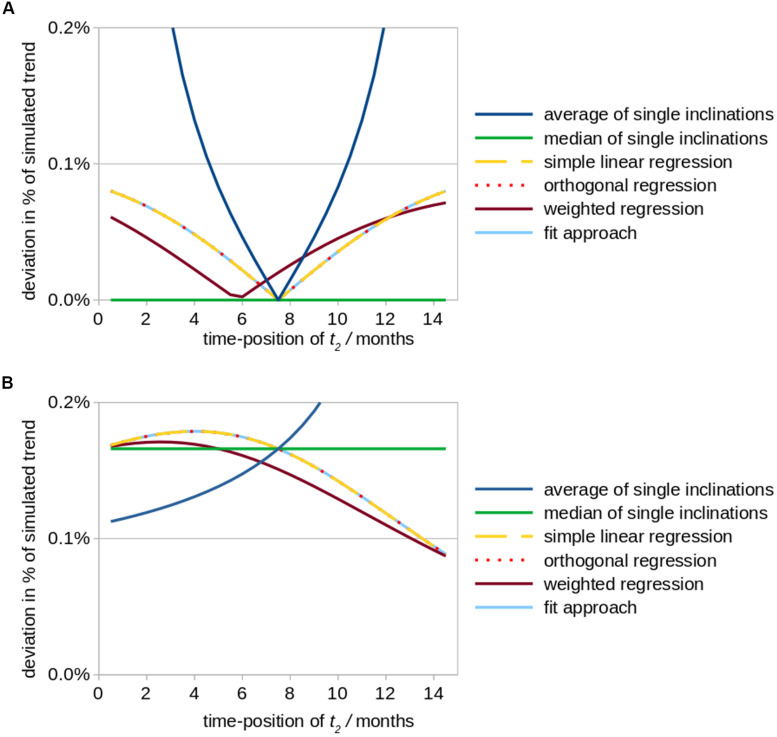
Simulated effects of a modified positioning of time point *t*_2_ (follow up 1) on calculations of striatal atrophy in Huntington patients. According to the underlying real world study (Müller et al., 2019), the total observation period amounted to 15 months and a monthly volume decrease of the striatum of 0.2% (in relation to its size at baseline) was assumed as original value of the overall linear trend. The position of time point *t*_2_ was varied between a position just behind baseline up to almost 15 months, while the total study duration of 15 months was kept constant. Aberrational data values at time point *t*_2_ (results shown in subfigure **(A)** and time point *t*_3_ **(B)** were simulated by an arbitrary artificial offset value of +0.3% (in relation to the striatum size at baseline, i.e., 8.3 cm^3^). The subfigures demonstrate the ensuing deviations (in percent of the original linear trend results) for different fit approaches. **(A)** Simulated aberration at *t*_2_ (follow-up 1). **(B)** Simulated aberration at *t*_3_ (follow-up 2).

In the following, the scenario of section “Simulations With Outliers and Time-Interval Dependency” was simulated, i.e., a striatal atrophy in Huntington’s disease with a linear volume loss of −0.2%/month:

(i) For the situation of a high quality measurement at *t*_3_ and a measurement at *t*_2_ of reduced quality, a follow-up 1 interval of 7.5 months (bisectioned observation period *[t_1_; t_3_]*) leads to a minimum of deviation of trend results from original values for *T*_*avg*_ as well as for *T*_*linReg*_ (as the artificial offset due to a low quality measurement at *t*_2_ is less or not considered); *T*_*median*_ provides no deviation at all for any time-position of *t*_2_ (Figure 2A). *T*_*orthReg*_, *T*_*linReg*_, and *T*_*fitApproach*_ reveal an almost equal course; *T*_*orthReg*_ and *T*_*linReg*_ are almost equal due to a comparatively low trend value (in the limit of trend = 0, *T*_*orthReg*_ and *T*_*linReg*_ would be equal). It has to be noted that especially the results for orthogonal regression *T*_*orthReg*_ highly depend on the amplitude of the trend. The position of the minimum of *T*_*weightReg*_ is depending on the weighting factors.

The results described above (i.e., with minimum deviations when time point 2 is exactly in the middle of the observation period) fits to the intuitive planning of identical time intervals and results from the fact that for identical time intervals the value of the first follow-up at time point 2 has no influence on the resulting trend (see section “Approaches Which Incorporate Timepoint 2 in the Trend Calculation”), even if aberrations occur. Out of the three fit approaches *T*_*avg*_, *T*_*linReg*_, and *T*_*median*_, *T*_*avg*_ is most sensitive to disturbances (aberrations), especially for non-equal time intervals. An alternative is to switch to a non-parametric calculation by using *T*_*median*_.

(ii) For the situation of a high quality measurement at *t*_2_ and a measurement at *t*_3_ of limited quality, similar deviations independent of the fit approach are observed. Only for a small interval between the two follow-up scans (*t*_2_ → *t*_3_), in *T*_*avg*_ higher deviations could be observed (Figure 2B). *T*_*orthReg*_, *T*_*linReg*_, and *T*_*fitApproach*_ reveal an almost equal course and (together with *T*_*weighted*_) reach a minimum deviation for (*t*_2_ → *t*_3_). *T*_*median*_ shows intermediate deviations.

Statistically, timing of the middle visit reveals that the deviation is minimized when follow-up 1 is shifted in time toward the time point of follow-up 2 (Figure 2). However, such a schedule might rather be realized for controls than for patients given that long-term observation periods in neurodegenerative diseases may be difficult due to a worsening of the physical and/or mental condition of the included patients with the consequences of decreased data quality and even dropouts.

## Discussion

In study design external conditions, and constraints, including time, budget, and feasibility, should be taken into account rather than searching for appropriate analysis tools with data in hand. With appropriate time interval planning, linear mixed effect models (Bernal-Rusiel et al., 2013) could be applied for data analysis as well as the further linear regression models (Rencher and Schaalje, 2007). The current theoretical study has been set up to induce a framework when longitudinal brain MRI studies in neurodegenerative diseases are planned for the specific but frequent case of one baseline and two follow-up scans. It is discussed which is the optimum model to be used as regression line for longitudinal trends. For the situation of one baseline and two follow-up MRI scans, the simulation of outliers on the one hand side reveals as optimal solution a simple schedule of identical time-intervals between baseline and follow-up scans which is the intuitive situation without any previous simulations. On the other hand, identical time-intervals constitute the paradox that follow-up 1 scan data do not influence the final trend results for either calculation method based on a linear model. This calls for the question if these follow-up 1 scans are indeed useful when the corresponding outcome parameters do not (or only partially) influence the trend results.

Thus, based on this theoretical framework, we provide a proposal how the time intervals in a longitudinal MRI study in neurodegenerative diseases could be conceptualized:

(1) We propose the time point for follow-up 1 to be shifted in time toward the time point of follow-up 2, that way considering that long-term observation periods in neurodegenerative diseases may be difficult due to a worsening of the physical and/or mental condition of the included patients. By this choice, it would be assured that follow-up 1 (*t*_2_) measurements influence the trend results and potentially stabilize them, which is particularly helpful if the last measurement [follow-up 2 (*t*_3_)] is of limited quality.
(2) We propose to avoid bisectioning the total observation period (baseline to follow-up 2) by follow-up 1. If bisectioning is unavoidable, the rater should be aware that the choice of the fit approach can significantly influence the trend results. An additional aspect is that it could be an alternative in the study schedule to skip the “median scans” (i.e., the follow-up 1 measurement in case of three visits) and to use the resources for a repeated follow-up 2 measurement.
(3) As a general consideration, it is recommended to perform a quality control of the imaging data prior to the fit procedure. Depending on the scan quality, it might be an option to discard single visit measurements (due to limited data quality) and to perform the fit with only two time points (instead of three time points) rather than attempting to increase the results quality by variation of the fit model. That way, it might be better to re-analyze a whole study with a higher quality requirement (on the data) than varying the fit approach at the analysis level.
Generally, it can be held that, in a given longitudinal MRI study with an even number of scans, the situation cannot occur that one scan does not influence the trend results for any time-interval planning. If, on the other hand, a study is planned with an odd number of scans and equal time-intervals, the “median” scans mostly do not influence the trend results.
(4) In neurodegenerative diseases, most participants are motivated and in a comparatively good physical condition at baseline. Thus, the time interval to follow-up is usually scheduled depending on the clinical evaluation of the disease progression and also the patients’ predicted physical condition (among further constraints). During the course of the study, the patients’ physical condition and their ability to comply with the requirements of neuroimaging are usually worsening, leading to missing data and (more important) to potentially reduced quality of scans at follow-up acquisitions. The latter effect leads to a variability of noise across subjects especially between controls and patients in longitudinal group comparisons which could be addressed by the application of a mixed effect model (if all recorded data passed quality control – see item 3).

Although concepts for longitudinal trend analysis are an issue of well-established research (Rencher and Schaalje, 2007), the technical complexity of modeling longitudinal data remains a topic of discussion and is, at least in certain aspects, incompletely understood and/or appreciated (Bernal-Rusiel et al., 2013). The application of linear regression methods in longitudinal neuroimaging studies has to be thoroughly considered and in general, the applicability of linear mixed effect models (Rencher and Schaalje, 2007) should be an analysis option under consideration, especially for the analysis of *ex post facto* data when the time intervals were already fixed (and equal). However, when a study with one baseline and two follow-up scans is conceptualized, a bisectioning of the observation time should be avoided in order to provide any analysis method the full power of accuracy. Advanced models such as least absolute shrinkage and selection operator (*Lasso*) (Tibshirani, 1996) which is a regression analysis method that performs both variable selection and regularization in order to enhance the prediction accuracy are generally oversized for solving linear regression of three data points. In addition to sophisticated models for harmonizing longitudinal multi-scanner imaging data (Beer et al., 2020), statistical analysis of longitudinal data with linear mixed effect models (Bernal-Rusiel et al., 2013), and normative estimates of longitudinal data (Fotenos et al., 2005), the current theoretical study has been set up to induce a framework when longitudinal brain MRI studies in neurodegenerative diseases are planned for the specific but frequent case of one baseline and two follow-up scans.

The key message of this theoretical study is that for the specific but frequent case of one baseline and two follow-up scans by bisectioning the total observation period (baseline to follow-up 2), follow-up 1 measurements do not influence the trend results or only in a weakened form. That way, we recommend to shift the follow-up 1 measurement toward the end of the study so that a bisectioning of the total time interval is avoided.

## Author Contributions

H-PM: study concept and design, simulations and interpretation of data, and drafting of manuscript. AB, GL, and H-JH: interpretation of data and critical revision of manuscript for intellectual content. JK: study concept and design, interpretation of data, and critical revision of manuscript for intellectual content. All authors contributed to the article and approved the submitted version.

## Conflict of Interest

The authors declare that the research was conducted in the absence of any commercial or financial relationships that could be construed as a potential conflict of interest.
